# From dysbiosis to defense: harnessing the gut microbiome in HIV/SIV therapy

**DOI:** 10.1186/s40168-024-01825-w

**Published:** 2024-06-21

**Authors:** Jason M. Brenchley, Sergio Serrano-Villar

**Affiliations:** 1 Barrier Immunity Section, Lab of Viral Diseases, NIAID, NIH, Bethesda, MA USA; 2 Department of Infectious Diseases, Hospital Universitario Ramon y Cajal, IRYCIS and CIBERInfec, Madrid, Spain

## Abstract

**Background:**

Although the microbiota has been extensively associated with HIV pathogenesis, the majority of studies, particularly those using omics techniques, are largely correlative and serve primarily as a basis for hypothesis generation. Furthermore, most have focused on characterizing the taxonomic composition of the bacterial component, often overlooking other levels of the microbiome. The intricate mechanisms by which the microbiota influences immune responses to HIV are still poorly understood. Interventional studies on gut microbiota provide a powerful tool to test the hypothesis of whether we can harness the microbiota to improve health outcomes in people with HIV.

**Results:**

Here, we review the multifaceted role of the gut microbiome in HIV/SIV disease progression and its potential as a therapeutic target. We explore the complex interplay between gut microbial dysbiosis and systemic inflammation, highlighting the potential for microbiome-based therapeutics to open new avenues in HIV management. These include exploring the efficacy of probiotics, prebiotics, fecal microbiota transplantation, and targeted dietary modifications. We also address the challenges inherent in this research area, such as the difficulty in inducing long-lasting microbiome alterations and the complexities of study designs, including variations in probiotic strains, donor selection for FMT, antibiotic conditioning regimens, and the hurdles in translating findings into clinical practice. Finally, we speculate on future directions for this rapidly evolving field, emphasizing the need for a more granular understanding of microbiome-immune interactions, the development of personalized microbiome-based therapies, and the application of novel technologies to identify potential therapeutic agents.

**Conclusions:**

Our review underscores the importance of the gut microbiome in HIV/SIV disease and its potential as a target for innovative therapeutic strategies.

## Background

During progressive human immunodeficiency virus (HIV) infection of humans and Simian immunodeficiency virus (SIV) of Asian macaque nonhuman primates (NHPs), ongoing viral replication and systemic inflammation contribute to the development of immunodeficiency, and most infections, if left untreated, will lead to individuals becoming susceptible to opportunistic infections. In people with HIV (PWH) treated with antiretroviral therapy (ART) and with sustained viral suppression, residual inflammation contributes to increased susceptibility to cardiovascular disease and development of some cancers [1]. Thus, there has been significant interest in understanding the causes of residual inflammation in PWH on ART.

The causes of inflammation in progressive HIV and SIV infections are multifaceted and include the following: co-infections [2], HIV or SIV themselves [3], and microbial translocation [4]. However, the degree to which each of these contributes to inflammation in PWH and SIV-infected Asian macaques on ART is unclear. Therefore, targeted therapeutic interventions have been considered to specifically reduce individual causes of inflammation in PWH and SIV-infected NHPs. Here, we review therapeutic interventions to reduce microbial translocation-induced inflammation in PWH or SIV-infected Asian macaques.

### Normal functions of the gut microbiome

The microbiota of the gut comprises approximately 10^14^ diverse bacterial organisms. Bacterial genes are at least 100 times greater than the human genome. This microbiota consists of approximately 1000 species of bacteria, belonging to two main groups: the Bacillota and the Bacteroidota. Humans have coevolved with the gut microbiota to maintaining a symbiotic relationship. Indeed, germ-free animals are more susceptible to infections and have reduced vascularity, digestive enzyme activity, muscle wall thickness, serum immunoglobulin levels, smaller Peyer’s patches, and fewer intraepithelial lymphocytes (reviewed in [5]). The beneficial roles of the gut microbiota are multifactorial and include immunological, structural, and metabolic functions [6].

The microbiota competes with pathogenic bacterial species and metabolizes dietary carbohydrates into short-chain fatty acids, a major nutritional source for the colonic epithelia [7]. Finally, most plasma cells secrete dimeric or oligomeric IgA, which can transcytose directly into the lumen. These antibodies are specific for luminal bacteria and viruses, suggesting that the microbiota shapes the specificity of luminal IgA [8]. Taken together, the microbiota provides invaluable functions to the host.

### The gastrointestinal tract in HIV/SIV infections

Early after HIV was discovered as the causative agent of acquired immunodeficiency disease syndrome (AIDS), significant gastrointestinal manifestations were noted [9]. The observed abnormalities including malabsorption and lymphocyte depletion led to the conclusion as follows: “The histologic findings suggest that a specific pathologic process occurs in the lamina propria of the small intestine and colon in some patients with the syndrome” [9]. This finding was incredibly insightful in its anticipation of subsequent discoveries. Over the years, we have learned that the mechanisms underlying the observed gut abnormalities are multifaceted. Indeed, there is overwhelming evidence to suggest that CD4 + T cells are disproportionately infected by the virus and depleted from the lamina propria of the gut compared to other anatomic sites of PWH and SIV-infected Asian macaques [10–16]. Moreover, progressive HIV/SIV infections lead to a massive depletion of CD4 + T cells in the gut of HIV/SIV-infected individuals, especially of CD4 + T cells producing the effector cytokines IL-17 and/or IL-22 [17–19]. Most epithelial cells in the body express the IL-22 receptor, and IL-22 is a growth factor supporting their survival and division. Moreover, IL-17 is a chemoattractant for neutrophils to anatomic sites where bacterial and fungal antigens are present [20–23]. Thus, preferential loss of lymphocytes producing these effector cytokines may provide mechanistic insights into damage to the structural barrier of the gut [20–23].

### Is there an HIV-associated dysbiosis?

In addition to the observed immunological perturbations being hypothesized to contribute to HIV-associated enteropathy, increasing information suggests that the composition of the gut microbiome can also contribute. Intestinal microbial dysbiosis — an imbalance in the composition of the individual bacteria within the gut microbiome — has been noted in PWH from different geographic locations [24–30], reviewed in [31]. However, dysbiosis of the bacterial gut microbiome in SIV-infected macaques is less obvious [32–36], suggesting confounding variables may contribute to the bacterial dysbiosis observed in PWH [37–39].

Indeed, alteration to the composition of the gut bacterial microbiome seems to contribute to many diseases, ranging from conditions which affect the gut itself [40] to neurological diseases [41]. Understanding mechanisms underlying bacterial dysbiosis observed in outbred populations of humans is complicated by the multiple confounding variables that can influence susceptibilities to particular diseases and the composition of the gut microbiome. Recent meta-analyses suggested that, in many cases, dysbiosis associated with diseases were attributed to confounding variables such as age, sex, body mass index, diet, and alcohol use [42]. Indeed, in microbiome analyses of PWH after controlling for risk factors associated with HIV acquisition, recent studies have demonstrated that the degree to which the microbiome becomes dysbiotic is predicted by the nadir CD4 + T-cell count [39]. These data suggest that the greater immune impairment associated with HIV infection contributes to dysbiosis.

Vertical transmission of a dysbiotic microbiome to HIV-exposed but uninfected infants may contribute to their increased morbitidies [43]. Research indicates variations in the microbiome composition, including fecal samples, between children born to HIV-positive mothers compared to those from HIV-negative mothers [44–46]. These microbial differences between HIV-exposed and unexposed children seem to mirror those found in their mothers and may be moderated by breastfeeding [47]. This suggests a possible transmission of the maternal microbiome, although it remains unclear whether these patterns are directly due to microbial transfer or influenced by environmental or lifestyle factors related to living with HIV, such as treatment with cotrimoxazole.

The impact of ART on the gut microbiome in PWH is another growing area of study, complicated by ART’s association with immunological improvement and the diversity of treatment combinations and indication bias. While some studies have begun to unravel the effects of ART on the microbiome, results remain varied. For example, in a longitudinal study in PWH starting ART based on integrase inhibitors or non-nucleoside reverse transcriptase inhibitors followed for 96 weeks, participants exhibited only minor changes in microbiota composition, regardless of the drug class [48]. Similarly, in another study in PWH starting ART at an advanced disease state, no substantial changes on gut microbiota composition were observed after 48 weeks of follow-up [49]. A recent study in Zimbabwe highlighted the role of geographical and baseline microbiota factors in ART’s effects, particularly in rural areas where ART with cotrimoxazole led to a reduction in alpha diversity after 24 weeks, with more significant changes in individuals having higher baseline diversity [50]. This suggests a potential direct drug effect on microbiome diversity. Indeed, a recent in vitro study elucidates an unexpected facet of ART — its antibacterial activity. Certain antiretroviral drugs exhibited in vitro antibacterial action against gut and vaginal commensal bacteria, particularly against multidrug-resistant strains [51]. Collectively, these findings indicate that ART’s influence on the gut microbiome is complex and requires further research to fully grasp its implications on the health of PWH.

### Are HIV-associated changes in the microbiome a harmful adaptation?

Recent advancements in profiling the functions of the microbiome, using omics technologies such as transcriptomics, proteomics, and metabolomics, have shed light on its relationship with inflammation. One study revealed that certain bacteria, including Bifidobacteria, exert functions with a crucial role in preventing mucosal defects and reducing microbial translocation, thereby aiding immune recovery. Others, like Succinivibrionaceae and Erysipelotrichaceae, buffer pro-inflammatory molecules and accumulate antiviral compounds, highlighting their potential to reduce inflammation and inhibit viral replication [52]. A study involving women with or at high risk of HIV focused on gut microbial species in relation to carotid artery atherosclerosis. It found associations between certain microbes like *Fusobacterium nucleatum* and carotid artery plaque, along with a microbial metabolite, imidazole-propionate (ImP), linked to plaque and pro-inflammatory markers [53].

Moreover, in a study combining metagenomics and metatranscriptomics, we found that the HIV-associated microbiota adapts to an inflamed environment by overexpressing oxidative stress resistance pathways but also shifting towards pro-inflammatory gram-negative bacteria enriched in lipopolysaccharide biosynthesis pathways and potentially pathogenic strains [54]. These findings suggest intricate interactions between bacterial metabolism and HIV immunopathogenesis. Furthermore, they pose a thought-provoking question: might what we perceive as dysbiosis actually represent an adaptation of the microbiome for the host, with the balance between beneficial and harmful adaptations contributing to the extent of persistent inflammation in PWH?

Additionally, the role of the microbiome in HIV elite controllers offers fascinating insights. A study investigating the fecal metabolome and microbiome in elite controllers revealed an enrichment of certain dipeptides and the genus *Prevotella*, suggesting a unique microbiome profile that might contribute to their natural control of HIV replication [55]. Another study found that ECs have a richer gut microbiota with distinct bacterial signatures and metabolic profiles, potentially playing a role in their ability to control HIV [56]. These findings highlight the potential of leveraging microbiome insights to develop novel therapeutic strategies aimed at enhancing immune control against HIV.

### Therapeutic interventions

**Fig. 1:**
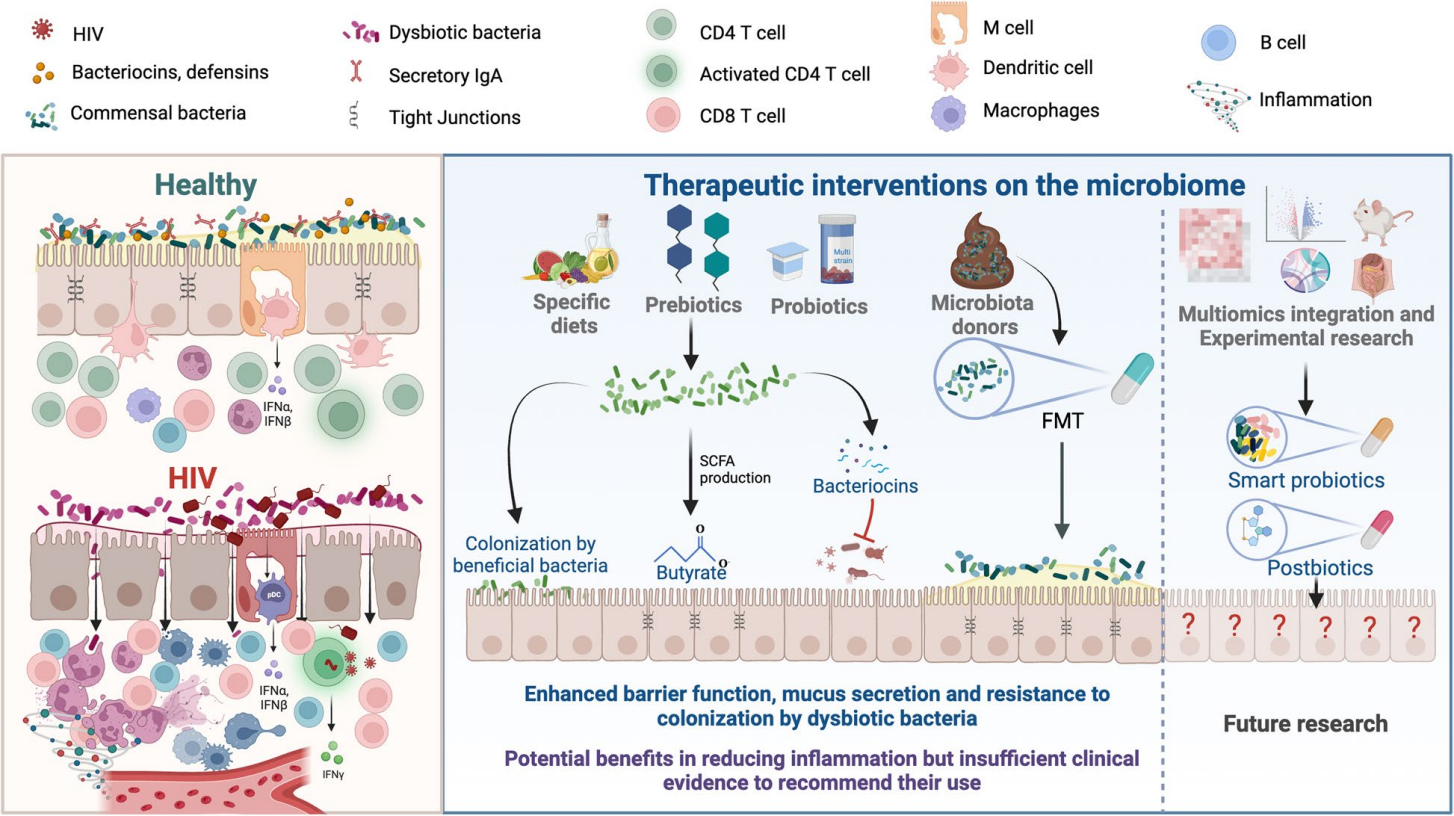
Therapeutic interventions targeting the microbiome in PWH. Left panel: GALT immune function and HIV disruption: healthy conditions, complex microbiome and balanced immune response in the GALT; HIV effects, altered microbiome, damaged enterocyte barrier, increased bacterial translocation, CD4 T-cell loss, CD8 T-cell expansion, neutrophil infiltration, local and systemic inflammation. Right panel: Intervention strategies and future directions: diet and prebiotics, Mediterranean diet and prebiotics promote growth of beneficial bacteria; probiotics, targeted addition of beneficial bacteria; FMT, replacing patient’s microbiota with a donor’s healthy microbiome; current state of evidence, interventions potentially reduce inflammation but lack sufficient clinical backing for recommendation; research prospects, future studies aim to produce targeted microbial consortia or postbiotics with heightened precision and efficacy

To better understand the degree to which dysbiosis (irrespective of the cause, disease, or underlying anatomic or physiological predispositions to disease) contribute to inflammation and pathology, interventional studies aimed at reducing dysbiosis are powerful tools. Here, we review microbiome-altering therapeutic interventions administered to PWH and SIV-infected NHPs, how these have resulted in microbiome alterations and influences they’ve had on immunity, and future directions for microbiome-directed therapies in PWH (Fig. 1).

### Antibiotics in SIV-infected NHPs and PWH

#### Previous research in NHPs

The most common family of therapeutic interventions aimed at altering the microbiome’s composition is antibiotics and is commonly used to reduce levels of bacteria thought to contribute to disease. Oral antibiotics significantly influence the composition of the gut microbiome [57, 58]. In SIV-uninfected NHPs, antibiotics have been experimentally given, followed by longitudinal assessment of the microbiome and immunological function. Enrofloxacin, cephalexin, paromomycin, and clindamycin all individually disrupt the composition of the gut microbiome, reduce levels of bacteria producing short-chain fatty acids, and induce some level of inflammation [58]. Importantly, the sources of NHPs that provide animals for biomedical sciences are diverse, and gut symptomologies are common in animals as they are transferred across facilities [57]. Indeed, we previously found significant alteration to the gut microbiome as NHPs are transferred from outdoor provisioned environments to indoor facilities [59]. In an attempt to ameliorate any alteration to the gut microbiome associated with acclimatizing to new environments, combinatorial use of enrofloxacin, paromomycin, fenbendazole, and azithromycin has been considered before study enrollment [57]. This multimodal, broad-spectrum, antibiotic use led to significant alterations to the gut microbiome that were fairly long-lived. Moreover, antibiotic use was associated with decreased gut inflammation and more consistent intrarectal SIV acquisition, but its influence on SIV disease progression was not analyzed.

Based on our current knowledge of microbial-immune interaction in PWH- and SIV-infected NHPs, significant alteration to the gut microbiome is anticipated to affect progressive HIV or SIV infections. Indeed, treatment of NHPs with vancomycin, an antibiotic against gram-positive bacteria, led to significantly decreased levels of bacteria within the Bacillota phylum (which produce energy-rich short-chain fatty acids) and increased levels of bacteria within the Pseudomonadota phylum (which contain highly motile and potentially pathogenic gram negative facultatively anaerobic rods) [60]. While repetitive vancomycin treatment maintained this dysbiotic microbiome and resulted in a significant reduction in the continuity of the epithelial barrier of the gut, surprisingly, there was no influence on canonical metrics of SIV disease progression [60] (Table 1). However, subsequent work demonstrated that vancomycin-induced dysbiosis was sufficient to increase the number of transmitted founder viruses after low-dose intrarectal SIV challenge in NHPs [61]. Another study in acutely SIV-infected macaques found that early treatment with rifaximin and sulfasalazine reduced microbial translocation and immune activation. Still, this effect diminished over time and was ineffective in chronically infected macaques [62].

**Table 1:** Main controlled studies in SIV and HIV with dietary interventions, prebiotics, postbiotics, symbiotics, and FMT

Population	Intervention	Study design and sample size	Outcomes	Ref
Rhesus macaques	Vancomycin	5 SIV + vancomycin 5 SIV control	Increased damage to GI tract epithelium	[60]
Rhesus macaques	Vancomycin	6 vancomycin before SIV challenge 6 control	Increased transmitter founder viruses after vancomycin	[61]
Pigtail macaques	Probiotics	5 (SIV + ART alone) 5 (SIV + ART + probiotics)	Increased GI tract CD4 + T-cell reconstitution	[63]
Pigtail macaques	Probiotics + IL-21	5 (SIV + ART alone) 5 (SIV + ART + probiotics + IL-21)	Increased GI tract CD4 + T-cell reconstitution, transient Th17 increases	[64]
Rhesus macaques	FMT	6 FMT + SIV 6 control SIV	Increased Th17 cells	[65]
Rhesus macaques	High-fat diet	8 HFD + SIV 52 historical SIV controls	Increased SIV disease progression	[66]
Pigtail macaque	High-fat diet	7 historical SIV controls 5 HFD + SIV 3 HFD	Increased SIV disease progression	[67]
Rhesus macaques	Butyrate	6 (SIV + ART alone) 7 (SIV + ART + butyrate)	Slight increase in jejunal H3 acetylation and modest microbiome alterations	[68]
PWH on ART	Fermented skimmed milk supplemented with *Lactobacillus rhamnosus* GG (108 cfu/mL), *Bifidobacterium animalis* subsp. Lactis B-12 (108 cfu/mL), and *Lactobacillus acidophilus* La-5 (107 cfu/mL) Duration: 8 weeks	Randomized, double-blind, placebo controlled trial *N* = 25 Probiotics: *N* = 12 Placebo: *N* = 7 Control: *N* = 6	Mild D-dimer, CRP, and IL-6 reduction. No changes in microbial translocation (LPS, sCD14) or T-cell activation markers	[69]
PWH on ART	*Saccharomyces boulardii* (two capsules three times a day or 6 × 10^7^ living bacteria) Duration: 12 weeks	Randomized,d ouble-blind, placebo controlled trial *N* = 35 Probiotics: *N* = 19 Placebo: *N* = 16	Decreased LBP No changes in inflammation markers or T-cell counts	[70]
PWH diagnosed with CD4 < 350 cells/µL or AIDS starting ART	A synbiotic mixture of prebiotics, *Saccharomyces boulardii*, oligonutrients, essential amino acids, omega-3 fatty acids Duration: 48 weeks	Randomized, double-blind, placebo controlled trial *N* = 59 Synbiotics: *N* = 32 Placebo: *N* = 27	No effects on primary (CD4 count, CD4/CD8 ratio) or secondary (T-cell activation, bacterial translocation, inflammation, or fecal microbiota structure) endpoints	[49]
PWH on ART with CD4 < 500 cells/µL and < 50 cells/µL increase 6 months before enrolment	*Lactobacillus casei* Shirota Duration: 12 weeks	Randomized, double-blind, placebo controlled trial *N* = 45 • Probiotics: *N* = 23 • Placebo: *N* = 22	No effects on CD4 count, CD4/CD8 ratio, T-cell activation, or sCD14	[71] 714,353
PWH on ART on ART with CD4 > 200 cells/µL	Probiotic visbiome For weeks 2–4, one sachet orally daily For weeks 4–26, one sachet orally twice daily	Randomized, double-blind, placebo controlled trial *N* = 73 • Probiotics: *N* = 42 • Placebo: *N* = 31	No effect on primary (sCD14) or secondary (markers of inflammation and gut permeability) enpoints	[72]
PWH on ART and CD4 < 350/ counts µL despite virologic suppression	Probiotic visbiome Duration: 48 weeks	Randomized, double-blind, placebo controlled trial • Probiotics: *N* = 18 • Placebo: *N* = 10	No effect on primary outcome (CD8 activation: HLA-DR + /CD38 + cells) or secondary (markers of inflammation, immune reconstitution, bacterial translocation, and gut permeability) endpoints CD4 + T-cell activation (HLA-DR +) increased	[73]
PWH on ART and CD4 < 500 cells/µL	Probiotics: *Lactiplantibacillus plantarum* and *Pediococcus acidilactici* Prebiotics: Pectin, inulin, oat, acacia, maltodextrin polydextrose, and partially hydrolyzed guar gum Duration: 6 months	Randomized, double-blind, placebo controlled trial 71 patients • Probiotics: *N* = 21 • Prebiotics + Probiotics: *N* = 32 • Placebo: *N* = 18	With synbiotics, slight increases of CD4 + counts and CD4/CD8 ratio, and decreased sCD14	[74]
PWH on ART and CD4/CD8 ratio < 1	Fecal microbiota transplant from three selected donors	Randomized, double-blind, placebo controlled trial *N* = 29 patients • FMT: *N* = 14 • Placebo: *N* = 15	Decreased in IFABP, increased alpha diversity, and increased Lachnospiraceae and Ruminococcaceae abundance No effects on CD4 counts, CD4/CD8 ratio, or other markers of inflammation and bacterial translocation	[75]
MSM on ART	Supplemented Mediterranean diet (SMD) with extra-virgin olive oil and nuts	Randomized, open controlled trial *N* = 60, according to SMD • High adherence: *N* = 30 • Medium adherence: *N* = 20 • Low adherence: *N* = 8	Improved lipid profiles and reduced immune activation with high adherence to SMD	[76]

#### Previous research in PWH

Rifaximin is a nonabsorbable antibiotic with established efficacy to reduce bacterial translocation and improves encephalopathy in patients with decompensated liver cirrhosis [77]. It has been considered as a possible modality to ameliorate persistent immune dysfunction in ART-suppressed PWH. However, in a trial that randomized PWH with poor immunologic recovery (CD4 counts < 350 cells/µL) to receive rifaximin or no therapy for 4 weeks, rifaximin elicited a minimal impact on bacterial translocation and CD8 + T-cell activation [78] and no effects on the rectal microbiome composition [79].

Cotrimoxazole, an antibiotic indicated to prevent opportunistic infections in PWH with advanced immune suppression, has also been proposed as beneficial in reducing bacterial translocation and inflammation. The COSTOP trial, which evaluated the impact of discontinuing cotrimoxazole on 174 ART-suppressed PWH, found that cotrimoxazol cessation increased inflammation markers (C-reactive protein and IL-6). However, since it did not significantly alter microbial translocation markers, this raises the question of whether cotrimoxazole’s impact on inflammation might be due to direct effects on prevalent infections, such as malaria and *Pneumocystis jirovecii*, in specific environments [80]. In children with HIV, maintaining cotrimoxazole has also been associated with decreased inflammatory markers but increased antibiotic resistance genes in their gut microbiota [81, 82].

Taken together, current evidence suggests that the composition of the gut microbiome influences susceptibility to SIV (and presumably HIV) acquisition but is less critical for disease progression. How the composition and function of the gut microbiome contribute to inflammation and comorbidities in PWH on ART is unclear, and further work is merited. Moreover, studies using antibiotics seem to suggest impact on the gut microbiome, while their use should be carefully thought of because of broad alterations to the microbiome and potential development of antibacterial resistance. An alternative approach would entail use of bacteriophage therapy, which has been shown to be more specific in targeting bacterial pathogens, and reducing antibacterial resistance might be considered [83].

### Prebiotics and probiotics

#### Previous research in NHPs

One of the most popular therapeutic interventions to increase the number of beneficial bacteria within the gut microbiome is the oral administration of live probiotic organisms [84]. While disparate in composition and quantity, probiotics are largely considered safe, but how they ultimately contribute to the gut microbiome and influence disease has been variably reported [85]. Secondly, providing particular nutrients to the microbiome has been considered to increase the growth of specific bacterial taxa thought to benefit the host [85]. In NHP models, probiotic administration to SIV-uninfected animals led to increased levels of T follicular helper cells and increased levels of IgA + B cells [86]. However, while the composition of the gut microbiome appears to influence vaccine-induced immunity [87], administration of probiotics before vaccination with HIV antigens did not result in improved vaccine-induced immunity [88].

In SIV-infected animals treated with ART, adjuvant therapy with probiotics increased the reconstitution of gut-resident CD4 + T cells [63], and if IL-21 cytokine therapy was also included, there were transient increases in the levels of IL-17-producing lymphocytes within gut tissues [64]. Indeed, the levels of *Lactobacillus* within the guts of SIV-infected animals were related to the frequencies of Th17 cells [89]. Finally, treating chronically SIV-infected animals with *Lactobacillus*-containing probiotics led to increased gut epithelial cell repair [90]. Importantly, although these therapies were hypothesized to exert their effects by modulating the gut microbiome, there were no consistent, observable alterations. Irrespective, all studies found some beneficial influence of probiotic administration on the immunological competency of SIV-infected animals. Therefore, interventional studies in humans were subsequently considered.

#### Previous research in PWH

HIV is a chronic inflammatory disease, where ongoing mucosal and systemic immune dysfunction, potentially affected by impairment of the intestinal barrier [91, 92] and the microbiome [24, 26, 52, 93], leads to persistent inflammation, thereby resulting in inadequate CD4 + T-cell recovery [94] and increasing the risk of mortality [95, 96]. For many years, efforts have been made to tackle the intricate defects in GALT linked to HIV, with the objective of reducing the long-term mortality risk associated with chronic inflammation using a range of interventions.

Numerous studies have explored the impact of prebiotics and probiotics in PWH on ART (Table 1). The first studies hinted at the potential benefit of these interventions to improve immune recovery. For example, in a controlled study in PWH on ART, the probiotic *Saccharomyces boulardii* decreased lipopolysaccharide-binding protein (LBP), a marker of bacterial translocation [70]. In another exploratory study, following a 6-week dietary supplementation with of prebiotics in PWH, we found modest reductions in bacterial translocation markers and T-cell activation markers, along with an improvement in thymic output and alterations in butyrate production [97]. While promising, most of these studies were exploratory, with limited sample sizes, assessed multiple outcomes, reporting effects only on some of them, and there were mixed results across studies [49, 69–74] (Table 1).

The two main controlled studies designed to assess the impact of probiotics or synbiotics on PWH on ART reported no differences in their primary outcomes [49, 98]. The PROMALTIA study explored the effects of nutritional intervention on 78 PWH starting ART at advanced stages, randomized to receive either PMT25341 (a mix of prebiotics, *Saccharomyces boulardii*, omega-3/6 fatty acids, and amino acids) or a placebo. The study found that the synbiotic mixture did not significantly impact CD4 + T-cell counts, CD4/CD8 ratios, or gut microbiota structure compared to the placebo. Ultimately, the study concluded that while ART alone provides clear immunological benefits in advanced HIV disease, synbiotics did not enhance these benefits [49]. While more notable effects on gut microbiota composition were found in vertically infected children and adolescents receiving PMT25341 [98], again, the intervention exerted no quantifiable influence on inflammation, microbial translocation, or mucosal integrity [99].

The A5350 phase II study examined the safety, tolerability, and effects on inflammation and intestinal barrier function of Visbiome Extra Strength (ES) on inflammation and intestinal barrier function in 73 participants on ART with CD4 + T-cell count > 200 cells/µL over 24 weeks. Again, despite some changes in the microbiome, including a decrease in Gammaproteobacteria, it showed no significant impact on systemic inflammatory markers [72]. Lastly, PROOV-IT, another exploratory-controlled study in PWH with less than 350 CD4 + T cells/mm^3^ despite viral suppression, found a consistent lack of effect using the same probiotic [73].

In conclusion, so far, the gut microbiota remains an elusive target for prebiotic and probiotic interventions in PWH. While some potential beneficial effects are described across studies, current evidence does not support using the current formulations of prebiotics or probiotics to decrease inflammation or enhance immune recovery in PWH. In addition, the absence of regulatory oversight from major bodies, such as the Food and Drug Administration (FDA) and the European Medicines Agency (EMA), poses further challenges in their investigation as therapeutic agents. Future research focusing on second-generation probiotics, which are characterized by their diversity, specific targeting, derivation from human microbiomes, and design for distinct health outcomes, informed by a comprehensive understanding of the microbiome’s role in human physiology, holds promise in advancing this field.

### Fecal transplants

#### Previous research in NHPs

As noted, probiotics have not been shown to significantly alter the composition of the gut microbiome, and they do not colonize for long periods of time [84]. An alternative approach to introducing very high numbers and species of bacterial taxa into the gut is fecal microbiota transplantation (FMT) [100]. FMT in patients suffering from *Clostridioides difficile* disease significantly reduces *C. difficile* levels, leading to clinical improvement [101]. Therefore, FMT has been considered a potential therapeutic intervention for progressive HIV/SIV infections. In SIV-infected NHPs, animals were treated with antibiotics before fecal transplantation. Antibiotic use altered the gut microbiome to a greater extent than FMT [65]. However, even though significant bacterial dysbiosis does not occur in progressive SIV infection of Asian macaques, FMT led to increased frequencies of IL-17 and IL-22-producing lymphocytes and reduced CD4 T-cell activation within the guts of SIV-infected animals.

#### Previous research in PWH

Given the significant dysbiosis associated with PWH and the high safety profile associated with the treatment, there has been more interest in conducting studies in PWH than in NHPs to mitigate chronic inflammation. The first study in PWH on ART explored the effect of a single FMT delivered by colonoscopy in six participants. While microbial engraftment was measurable in this study, not all subjects experienced engraftment, which was more pronounced among the individuals with lower alpha diversity at baseline, and the effect tended to disappear during the 24-week follow-up [102]. In another uncontrolled study, six patients on ART received six weekly doses of lyophilized fecal microbiota from healthy donors, proving to be a safe and well-tolerated intervention. While microbiota diversity increased in participants with initially low diversity, the metagenomic analysis revealed no consistent changes, suggesting future trials should focus on PWH with more significant inflammation, gut damage, or dysbiosis [103].

In our pilot placebo-controlled study involving 30 PWH on ART, we asked whether repeated oral FMT could attenuate HIV-associated microbiota changes and systemic inflammation [75]. Because a hallmark of the HIV-associated microbiota is a depletion of butyrate-producing bacteria (Lachnospiraceae and Ruminococcaceae families) [31], we specifically searched for donors with enrichment for butyrate in their feces. Weekly FMT was safe and led to increased microbiota diversity and temporary engraftment of donor microbiota, with greater engraftment observed in those with recent antibiotic use. Additionally, FMT showed potential in reducing intestinal damage, as indicated by lower intestinal fatty acid-binding protein (IFABP), a biomarker predicting mortality in PWH on ART for long-term ART [96]. Collectively, studies in PWH indicate that FMT can lead to some changes in gut microbiota and encourage further exploration of strategies to address immune dysfunction by targeting the microbiota.

### Postbiotics and live biotherapeutic products (LBPs)

If we can conclusively show that alterations in the microbiota lead to tangible clinical or immunological benefits, the subsequent step would naturally involve identifying the specific microbiota components responsible for these effects. This knowledge would pave the way for creating targeted products, like specific bacterial consortia or the emerging field of postbiotics. A prime example of progress in this area is the treatment of *C. difficile*. Following the proven effectiveness of microbiota transplants, live biotherapeutic products (LBPs)—live microorganisms to modulate the microbiome—have proved efficacy and have gained approval to become commercially available [104, 105]. There are immense research opportunities in HIV.

For example, short-chain fatty acids (SCFAs) produced by certain gut bacteria exhibit anti-inflammatory properties [106]. A postbiotic intervention that might be hypothesized to decrease intestinal permeability and reduce microbial translocation-induced inflammation is butyrate [68]. As noted, this short-chain fatty acid is an important microbiome-produced metabolite that provides energy to the epithelium. However, high doses of butyrate in ARV-treated, SIV-infected, NHPs were insufficient to reduce residual inflammation in these animals. Indeed, very few immunological benefits were noted after butyrate supplementation, possibly due to butyrate absorption within the small intestine. A less absorbed, but bioactive compound such as tributyrin or probiotic organisms which produce high levels of butyrate may provide clinical benefits [107].

PWH display lower levels of butyrate-producing bacteria, such as *Roseburia intestinalis*, which correlates with increased systemic immune activation and HIV replication [97, 108]. Adding butyrate exogenously in vitro reduced T-cell activation and HIV replication, suggesting that butyrate modulates gut immune responses in HIV [108]. We previously observed fecal butyrate increases following prebiotic dietary supplementation, which correlated with amelioration of the inflammatory biomarkers soluble CD14 and high-sensitivity C-reactive protein [97]. However, in a randomized controlled trial investigating the effects of vitamin D and phenylbutyrate supplementation — a butyrate derivative — on gut-derived inflammation and microbiota composition PWH, no effects on gut-derived inflammatory markers or the microbiota in treatment-naïve PWH were found [109].

Therefore, despite the links between butyrate and microbial translocation-induced inflammation shown in studies on SIV-infected NHP and PWH, the role of butyrate as a postbiotic in PWH remains to be proven. Further research in the postbiotis field is needed.

### Dietary interventions

#### Previous research in SIVs

In addition to oral use of probiotics, other medicinal food and other dietary interventions have been considered for potential alteration of the gut microbiome and physiology. Diets high in fat and sugars alter the composition of the gut microbiome and increase gut inflammation [110]. However, while a high-fat diet significantly increased inflammation in NHPs, only modest alteration to the gut microbiome was observed [66, 67]. Importantly, inflammation associated with the high-fat diet dramatically accelerated progressive SIV infection of NHPs, suggesting that novel therapeutic interventions aimed at reducing obesity-associated inflammation may improve the prognosis of PLWH.

#### Previous research in PWH

In PWH, dietary interventions, particularly the Mediterranean diet supplemented with nuts and olive oil, have shown promise in improving gut microbiota, reducing inflammation, and potentially impacting microbial translocation. A study examined 102 PWH over 12 weeks, comparing those on a supplemented Mediterranean diet with extra-virgin olive oil and nuts to a control group on a regular diet. After classifying individuals by their adherence to the Mediterranean diet, those highly adherent exhibited improved lipid profiles and reduced immune activation. Notably, gut microbiota diversity and richness increased in the high-adherence group, with significant correlations between Succinivibrio and Bifidobacterium abundances and Treg cell levels [76].

In an observational study, we identified two dietary patterns among PWH: Mediterranean like and Western like. Participants with a Mediterranean pattern showed higher Lachnospira abundance and lower levels of the inflammatory biomarkers D-dimer and soluble tumor necrosis factor receptor 2 (sTNFR2), two independent predictors of mortality [96], compared to the Western-diet fed group. The Mediterranean pattern was also associated with lower Erysipelotrichaceae abundance and inflammatory markers, indicating its potential to reduce inflammation-related processes in PWH [111].

In summary, despite the limited availability of clinical trials evaluating the effects of dietary interventions on gut microbiota and inflammation in PWH, existing data suggest that healthier dietary choices can positively alter the microbiota and diminish inflammation in PWH. This aligns with findings from extensive research conducted in the general population [112].

### Other interventions

Beyond the above interventions, there are other gut-oriented strategies to reduce inflammation in PWH. However, most studies have yielded negative results. For example, mesalamine, an anti-inflammatory agent effective in ulcerative colitis, was tested in a placebo-controlled trial to assess its impact on abnormal immune activation in PWH. The study, involving 33 PWH on ART with incomplete immune recovery (CD4 < 350 cells/mm^3^), showed no significant change in various immune markers, including canonical markers of T-cell activation (CD38 + HLA-DR + CD4 + and CD8 + T cells) in blood and rectal tissue, or in various soluble markers of inflammation, after 12 weeks of mesalamine treatment [113].

## Challenges and limitations

In the field of SIV/HIV microbiome research, we are confronted with numerous complexities. Central to these challenges is the complex interplay between the microbiome and the immune system, especially in the context of HIV. How variable is the microbiome across and within individuals? What are the fundamental characteristics of a “healthy” microbiome, and how do these vary across different populations influenced by genetics, lifestyle, and environmental factors [114]? How can we diagnose “dysbiosis”?

One significant obstacle is the individual variability in microbiome composition. This not only raises questions about the standardization of study conditions but also about the generalizability of research findings [115]. The influence of external factors like sexual practices, geographical location, and dietary habits adds further complexity. How do these factors interplay with HIV to affect the microbiome?

The scarcity of longitudinal studies poses another hurdle. What are the long-term risks of modifying the microbiome, and do the effects of interventions last over time? Ethical considerations, especially concerning invasive procedures for detailed microbiome analysis, further complicate these efforts. Moreover, interactions between ART and the gut microbiome modulations, together with the variability in ART regimens, ART length, and immune recovery, add another layer of complexity. Technological and analytical limitations, particularly in handling highly multidimensional data, are especially relevant in proteomics and metabolomics studies, essential for understanding the microbiome at a functional level.

Intervention studies in the SIV/HIV microbiome area face distinct challenges. The market is flooded with prebiotics and probiotics, often lacking sufficient research-backed evidence. How can we ensure the pharmacological properties of these products? There is also significant heterogeneity in dosage, duration, and measured outcomes across studies. What standardized outcome definitions and designs can we implement to ensure reliable evaluations and follow-ups?

FMT presents its own set of uncertainties. Which donor microbiota profiles are optimal for FMT in PWH? Do FMT donor profiles need to be individualized? Is an antibiotic conditioning regimen necessary for a more impactful microbiota modification? The lack of consensus on these issues points to the need for a more standardized and coordinated approach.

In summary, addressing these challenges calls for an interdisciplinary strategy, integrating advancements in technology and analytical methodologies. The list of challenges and potential solutions can be found in Tables 2 and 3, highlighting the need for collaborative efforts to realize the potential of microbiome research in HIV management.

**Table 2:** Challenges and solutions for limitations in SIV/HIV microbiota research

Research barrier	Specific challenge	Proposed solution
Complexity of microbial interactions	Difficulty in understanding interactions, causality vs. correlation issues	Enhanced multidisciplinary studies, advanced computational modeling coupled with experimental validations
Incomplete definition of a healthy microbiota	Lack of consensus on “healthy” microbiome, variability across populations	Standardized guidelines for microbiota research, global collaborative research
Individual variability in microbiome	Inter-individual differences, standardization issues	Personalized medicine approaches, large-scale population studies
Confounding effects of sexual practices	Isolating effects of HIV beyond confounders	Targeted research on sexual behavior impacts, controlled study designs
Confounding effects of geographical and dietary factors	Regional microbiome variation, generalization challenges	Cross-cultural studies, inclusion of diverse dietary patterns in research
Interactions with ART	ART-microbiome interplay, regimen variability	Comprehensive ART-microbiome interaction studies, standardized ART protocols
Limited longitudinal data	Scarcity of long-term studies, safety and efficacy assessments	Longitudinal study designs, posttreatment follow-up studies
Ethical and clinical trial constraints to study microbial interactions in tissue	Ethical concerns with invasive studies, clinical trial complexities	Develop innovative noninvasive techniques to sample tissues
Lack of standardized methodologies	Methodological differences, standardization need in proteomics and metabolomics	Development of universal protocols, collaborative methodological research
Technological and analytical challenges	Limitations in technology to understand the microbiota, data analysis complexities	Investment in advanced analytical tools, collaboration with data scientists
Generalizability of findings	Difficulty in applying findings broadly, microbiome composition variations	Multicentric studies, inclusion of diverse participant profiles
Interdisciplinary integration	Need for integrated research, coordination challenges	Interdisciplinary research programs, collaborative platforms
Funding and resource allocation	Limited funding, resource prioritization	Advocacy for increased funding, diversification of funding sources

**Table 3:** Challenges and solutions for limitations in current intervention studies in SIV/HIV microbiome research

Limitation category	Specific limitation	Proposed solution
Prebiotics and probiotics market proliferation	Marketed without research evidence, efficacy and safety variation	Stricter regulations, evidence-based marketing
Heterogeneity in intervention studies	Dosage and duration heterogeneity, outcome inconsistency	Standardized intervention protocols, unified outcome measures
FMT challenges	Donor profile uncertainty, antibiotic preconditioning consensus	Donor profile databases, clinical trials assessing preconditioning antibiotic regimens
Effectiveness assessment and localization	Stool vs. tissue microbiota correlation, impact assessment challenges	Advanced diagnostic tools, in-depth impact studies
Small sample sizes and short study duration	Limited sample size, short study duration	Larger, multicenter trials, extended study periods
Study design	Lack of standardized protocols, study design variability	Protocol development committees for microbiota interventions

## Future directions

Future directions in SIV/HIV microbiome research must focus on unexplored domains while refining our understanding of established concepts. The role of the microbiome in HIV transmission and its influence on comorbidities is fertile ground for research.

A granular understanding of microbiome-immune interactions is essential. Current knowledge is rudimentary, and future research should detail these interactions to design more effective interventions. This effort demands a meticulous examination of the molecular dialogues between microbial communities and immune responses. Such insights are vital for developing targeted therapeutic strategies, particularly in modulating immune responses and controlling inflammation.

Personalized microbiome-based therapies represent another promising frontier. The inter-individual variability of the microbiome suggests that tailored therapeutic approaches could be more effective than one-size-fits-all solutions. Research must pivot towards understanding individual microbiome profiles and their interactions with the immune system, paving the way for customized treatment protocols. For example, if we are able to identify detrimental bacteria, we should design personalized strategies to extract them from the microbiome. Exploring the potential of bacteriophage therapy to selectively target detrimental bacteria within the microbiota of PWH offers a promising avenue. This approach, if harnessed effectively, could minimize the risk of inducing antibiotic resistance genes, compared to traditional antibiotics.

Defining outcomes is another crucial area. Precise parameters for inflammation, immune recovery in immunological nonresponders, HIV transmission, and the management of comorbidities need establishment. These outcomes should not only guide research but also inform clinical practice, aiding in developing more effective treatment strategies.

Technological advancements are pivotal in transitioning beyond current interventions like FMT and pre-/postbiotics. The search for potential therapeutic agents from the microbiome demands not just sophisticated technologies but also a focus on exploring its “dark matter.” This includes the extensive virome and genome of uncultivated taxa, which are functionally significant yet remain largely unknown [116]. Investment in advanced analytical tools, high-throughput sequencing, and bioinformatics is essential. Collaboration with data scientists and bioinformaticians, followed by experimental validation of the detected hits, will facilitate handling and interpreting the complex data sets generated by these studies.

## Conclusions

Multiple recent studies have highlighted the complex interplay between the microbiome and SIV/HIV, emphasizing its significant implications for disease progression and treatment. We have gained substantial insights into how disruptions in the microbiome contribute to bacterial translocation and inflammation, paving the way for innovative therapeutic interventions.

However, this field faces notable challenges. The diversity in microbiome composition among individuals, shaped by genetics, lifestyle, and environment, complicates the development of universal treatment protocols. Confounding variables, such as diet, sexual practices, immune status, and ART, add layers of complexity, necessitating a subtle methodological approach. The requirement for long-term studies to understand the enduring impacts of microbiome alterations is clear, as is the ethical responsibility in conducting such research.

Despite these obstacles, the potential of microbiome-based therapies in HIV treatment remains promising. Exploring prebiotics, probiotics, and FMT has introduced new possibilities, though their efficacy and safety continue to be actively researched. The emergence of postbiotics and LBPs represents a new frontier in leveraging the microbiome to achieve better health outcomes in PWH. Dietary interventions have also shown promise in altering the microbiome and reducing inflammation, offering a less invasive method to modulate the microbiome. The efficacy of these interventions, along with other innovative strategies like the use of GLP-2 agonists, highlights the value of a multidisciplinary approach in addressing HIV’s complexities.

Looking ahead, the future of this field depends on integrating advanced technological and analytical methods with collaborative, interdisciplinary efforts. Such a comprehensive approach is essential to unlocking the full potential of microbiome research in HIV treatment. As we advance, awareness of the current challenges and limitations highlighted in this review will lay a more robust foundation for future studies, steering us towards more effective and personalized HIV treatments (Fig. 1).

## Data Availability

No datasets were generated or analysed during the current study.
